# Detection of substantial numbers of latent tuberculosis and positive hepatitis B serology results in rheumatology patients preparing to receive intensified immunosuppressive therapy in a low-prevalence country: why screening still matters

**DOI:** 10.1007/s10067-025-07350-x

**Published:** 2025-02-25

**Authors:** Martin Feuchtenberger, Magdolna Szilvia Kovacs, Axel Nigg, Arne Schäfer

**Affiliations:** 1 MVZ MED BAYERN OST, Burghausen, Germany; 2 Medizinische Klinik und Poliklinik II, University Hospital Würzburg, Würzburg, Germany; 3 Diabetes Zentrum Mergentheim, Bad Mergentheim, Germany

**Keywords:** Disease-modifying antirheumatic drugs, Hepatitis B, Hepatitis C, Latent tuberculosis infection, Rheumatology, Screening

## Abstract

**Introduction /objectives:**

International guidelines recommend screening for latent tuberculosis infection (LTBI) and chronic viral hepatitis infections before initiating intensified immunosuppressive therapy. We assessed the prevalence of positive screening tests for LTBI, hepatitis B virus (HBV), and hepatitis C virus (HCV) in patients screened at a large rheumatology outpatient center in Germany.

**Method:**

This retrospective cohort study used electronic health records from adult rheumatology patients. The presence of LTBI was evaluated by chest X-rays, patient medical history/self-report, and QuantiFERON®-TB Gold Plus (QFT) interferon-gamma release assays. Antibodies to HBV core antigen (anti-HBc) and HCV were used to assess HBV and HCV, respectively. Statistically significant associations were evaluated by Fisher exact tests.

**Results:**

Of 697 screened patients with a rheumatological condition (61.3% female, mean age 60.0 years), 132 (18.9%) patients were positive for LTBI (99 [14.2%]), anti-HBc (39 [5.6%]), or anti-HCV (3 [0.4%]). Nine patients had more than one positive result; different infections were not significantly associated. QFT detected the most LTBI cases (59.5%) followed by patient report/history (42.4%) and chest X-rays (17.2%). Although most patients (83.8%) were positive on only one test, associations among LTBI tests were statistically significant. Biologic disease-modifying antirheumatic drug (bDMARD) use was lower in patients with a positive LTBI screening result compared with all screened patients (73.7% vs 86.4%) and targeted synthetic DMARD (tsDMARD) use was higher (10.1% vs 5.9%).

**Conclusions:**

Almost one-fifth of rheumatology patients preparing to initiate intensified immunosuppressive therapy have positive results on screening tests for LTBI or show evidence of exposure to HBV or HCV. These findings support the need for careful screening, even in “low-prevalence” countries.

**Table:** 

**Key Points** • *Almost 20% of rheumatology patients preparing for intensified immunosuppressive therapy were positive on screening tests for latent tuberculosis infection (LTBI) or showed evidence of exposure to hepatitis B virus (HBV) or hepatitis C virus (HCV).* • *All three LTBI assessments (chest X-ray, patient medical history/self report, and interferon-gamma release assay) were important in determining LTBI status and seldom overlapped.* • *Positive results for LTBI, HBV, and HCV were not significantly associated.* • *Compared with all screened patients, biologic disease-modifying antirheumatic drug (DMARD) use was lower in patients with a positive LTBI result and targeted synthetic DMARD use was higher, but this result must be interpreted with caution.*	

## Introduction

In patients with latent bacterial or chronic viral infections, treatment with intensified immunosuppressive medications, including those commonly used in rheumatology such as glucocorticoids and disease-modifying antirheumatic drugs (DMARDs), can result in reactivation of infection [1]. Although other microorganisms may also be involved, some of the most common and clinically important pathogens involved in reactivation are *Mycobacterium tuberculosis*, the cause of latent tuberculosis (TB) infection (LTBI) [2, 3], hepatitis B virus (HBV) [4, 5], and hepatitis C virus (HCV) [6]. German guidelines recommend screening for LTBI and HBV before initiating biologic DMARD (bDMARD) or targeted synthetic DMARDs (tsDMARDs) [7], and recent European Alliance of Associations for Rheumatology (EULAR) guidelines extend these recommendations to include HBV and HCV screening prior to initiation of any immunosuppressive therapy (bDMARDs, tsDMARDs, conventional synthetic DMARDs [csDMARDs], immunosuppressants, or glucocorticoids), and consideration of LTBI screening in patients with increased risk for LTBI prior to starting csDMARDs, immunosuppressants, or glucocorticoids (in addition to screening prior to b/tsDMARD initiation) [8]. American College of Rheumatology (ACR) guidelines also recommend screening for LTBI in all patients preparing to initiate bDMARDs or tsDMARDs [9–11]. Although ACR guidelines address the management of patients with viral hepatitis, they do not specifically comment on HBV or HCV screening practices.

For LTBI testing, EULAR guidelines recommend chest X-rays and interferon-gamma release assays (IGRA), which are preferred over tuberculin skin tests where available because IGRA is less affected by immunosuppressive treatment and Bacille Calmette-Guérin (BCG) vaccination [8, 12, 13]. ACR guidelines recommend an initial IGRA or tuberculin skin test followed by a chest X-ray in patients with positive results [10]. Recommended HBV screening includes tests for hepatitis B surface antigen (HBsAg) to identify HBV carriers and tests for antibodies to the hepatitis B core antigen (anti-HBc) to identify patients with HBV infections at risk for reactivation [8]. HCV screening involves tests for anti-HCV antibodies.

Germany is considered to be a low prevalence country for TB, HBV, and HCV. For TB, low prevalence has been defined as < 100 reported cases per 1 million population [14]; the most recent German data indicate that there were 49 reported TB cases per 1 million population in 2022 [15]. Low HBV prevalence is considered < 2% of the population positive for HBsAg [16] and current data indicate an HBsAg prevalence of 0.3 to 0.7% in Germany [17]. There is no widely-accepted definition of low prevalence for HCV, but the estimated rate of 0.2% to 1.9% reported in Germany for anti-HCV [17] compares favorably with other European countries [18].

Despite these overall low prevalence rates, some subpopulations in Germany have higher rates of TB and hepatitis infection [19, 20]. However, there is minimal current information on the frequency of positive LTBI, HBV, and HCV findings in rheumatology patients initiating intensified immunosuppressive therapy during routine clinical practice in Germany. The goal of our study was to determine the prevalence of positive screening tests indicating possible prior exposure to pathogens with the potential for reactivation and evaluate associations among them. We also assessed whether different LTBI testing methods yielded concordant results and evaluated descriptive data on subsequent therapy choice in patients with or without positive LTBI findings.

## Methods

### Study design and objective

This retrospective cohort study was based on electronic health record (EHR) data collected from patients 18 years of age or older who presented in person to a single, large secondary care center specializing in rheumatology between July 2011 to February 2024 and were preparing to initiate intensified immunosuppressive therapy with bDMARDs, tsDMARDs, or high-dose glucocorticoids (≥ 1 mg per kg body weight). All patients included in these analyses had a primary diagnosis of a rheumatology condition as defined by International Classification of Diseases (ICD)−10 codes. Patients with active TB or whom had previously undergone treatment for LTBI were excluded from the dataset. No other inclusion or exclusion criteria were applied.

In addition to data on patient characteristics and screening test results (see below), data on subsequent therapy in the time period immediately following the screening tests (a time span of a few weeks) were collected. Data on treatments following this period were not analyzed. Due to differences in clinical context and dosing regimens, structured subgroup data for patients receiving subsequent treatment with high-dose glucocorticoids were not available.

The study was approved by the Institutional Review Board of Würzburg University with a waiver for individual patient consent given the retrospective design and use of de-identified patient data (#207/21-me). All research activities were conducted in accordance with the ethical principles outlined in the Declaration of Helsinki.

The objective of this study was to determine the prevalence of positive findings of LTBI, anti-HBc, and anti-HCV in rheumatology patients preparing to initiate intensified immunosuppressive therapy. Additionally, the study aimed to explore associations between positive screening test results for different latent or chronic infections and to assess the concordance among various LTBI testing methods.

### Screening tests

Screening for LTBI included evaluation of chest X-rays, patient medical history of TB infection or self-reported potential TB exposure, and QuantiFERON®-TB Gold Plus (QFT; QIAGEN, Hilden, Germany) IGRA. All three LTBI tests were performed on the same day for each patient. Anti-HBc tests utilized Elecsys® Anti-HBc II (Roche Diagnostics, Mannheim, Germany). Anti-HCV tests utilized Elecsys® Anti-HCV II (Roche Diagnostics, Mannheim, Germany). All serologic tests were conducted per the manufacturer’s instructions and using the manufacturer’s recommended cut-offs for positive vs negative results.

In cases of abnormalities in the chest X-ray, such as fibrotic scarring or calcified nodules, further imaging techniques were employed, including chest computed tomography, and, if necessary, bronchoalveolar lavage, biopsy, and other advanced diagnostic methods. If no alternative cause for the observed changes could be identified, the findings were considered indicative of LTBI. As the focus of our study was the prevalence of positive screening results, these additional analyses were not included in our data.

For patients with positive results on hepatitis screening tests, further testing for HBsAg or viral nucleic acids was conducted on an individual basis as determined by the treating clinician. These subsequent tests were not included in our database.

Standardized re-screening tests were not performed as part of routine procedure, as German guidelines recommend that re-screenings be reserved for specific situations (e.g., particular risk exposures or residence in high-risk areas).

### Statistical analysis

No calculations were made for the optimal sample size in this exploratory study; all patients who met entry criteria were included. This study is a retrospective analysis, utilizing an existing dataset to explore general trends and associations. The focus was on generating hypotheses and insights for future research rather than on confirming pre-specified hypotheses with controlled error rates.

Descriptive statistics, including mean, standard deviation (SD), and proportions, were used to summarize the demographic and clinical characteristics of the study population (age, gender, and specific rheumatologic diagnoses). The prevalence was calculated as the proportion of patients with positive test results among the total number of patients screened.

Fisher’s exact test was used to assess the statistical significance of associations between different screening results (e.g., LTBI and anti-HBc, LTBI and anti-HCV). Correlations between different LTBI screening methods (QFT, patient history/self-report, and chest X-rays) were analyzed using Spearman’s rank correlation coefficient. This non-parametric measure was chosen due to the ordinal nature of the data.

P values < 0.05 were considered statistically significant for all tests. All analyses were two-sided. No alpha adjustment or Bonferroni correction was applied in this analysis, as the aim of this exploratory study was to identify trends and potential associations rather than to test specific, directed hypotheses. The retrospective design and the focus on providing an overview and understanding of patterns in the data further justify this approach. Overly stringent corrections could obscure meaningful trends that warrant further investigation in future studies. Statistical analyses were conducted using SPSS for Windows, version 29.0 (IBM Corp., Armonk, NY). Microsoft Excel (Version 16.8.7) and GraphPad Prism (Version 10.2.3) were used to conduct simple analyses.

## Results

This retrospective analysis included 697 patients with a rheumatological condition preparing to initiate intensified immunosuppressive therapy. The majority (61.3%) were female and the mean (SD) age was 60.0 (15.8) years. Slightly over half of patients (55.5%) had a diagnosis for rheumatoid arthritis (RA) and the remainder were distributed among other rheumatological disorders (Table 1). Fewer than 10% of patients (9.6%) knew their TB vaccination status and 3.2% had a known TB vaccination.

**Table 1:** Baseline characteristics of rheumatology patients undergoing screening prior to immunosuppressive therapy. Data are reported as n (%) unless otherwise indicated

Characteristic	All patients	Patients with a positive LTBI screening result
N	697	99
Female	427 (61.3%)	58 (58.6%)
Mean (SD) age, years	60.0 (15.8)	64.0 (13.8)
Diagnosis		
Rheumatoid arthritis	387 (55.5%)	62 (60.6%)
Peripheral SpA	145 (20.8%)	15 (15.2%)
Axial SpA	90 (12.9%)	12 (12.1%)
Vasculitis	37 (5.3%)	7 (7.1%)
Other inflammatory conditions^a^	23 (3.3%)	1 (1.0%
Other arthritis	9 (1.3%)	1 (1.0%)
Connective tissue disease	6 (0.9%)	1 (1.0%)
Tuberculosis vaccination		
Unknown	630 (90.4%)	84 (84.8%)
No	45 (6.5%)	9 (9.1%)
Yes	22 (3.2%)	6 (6.1%)

^a^Including polymyalgia rheumatica

bDMARD, biologic disease-modifying antirheumatic drug; LTBI, latent tuberculosis infection; SD, standard deviation; SpA, spondyloarthritis; tsDMARD, targeted synthetic disease-modifying antirheumatic drug

### Screening results

**Fig. 1:**
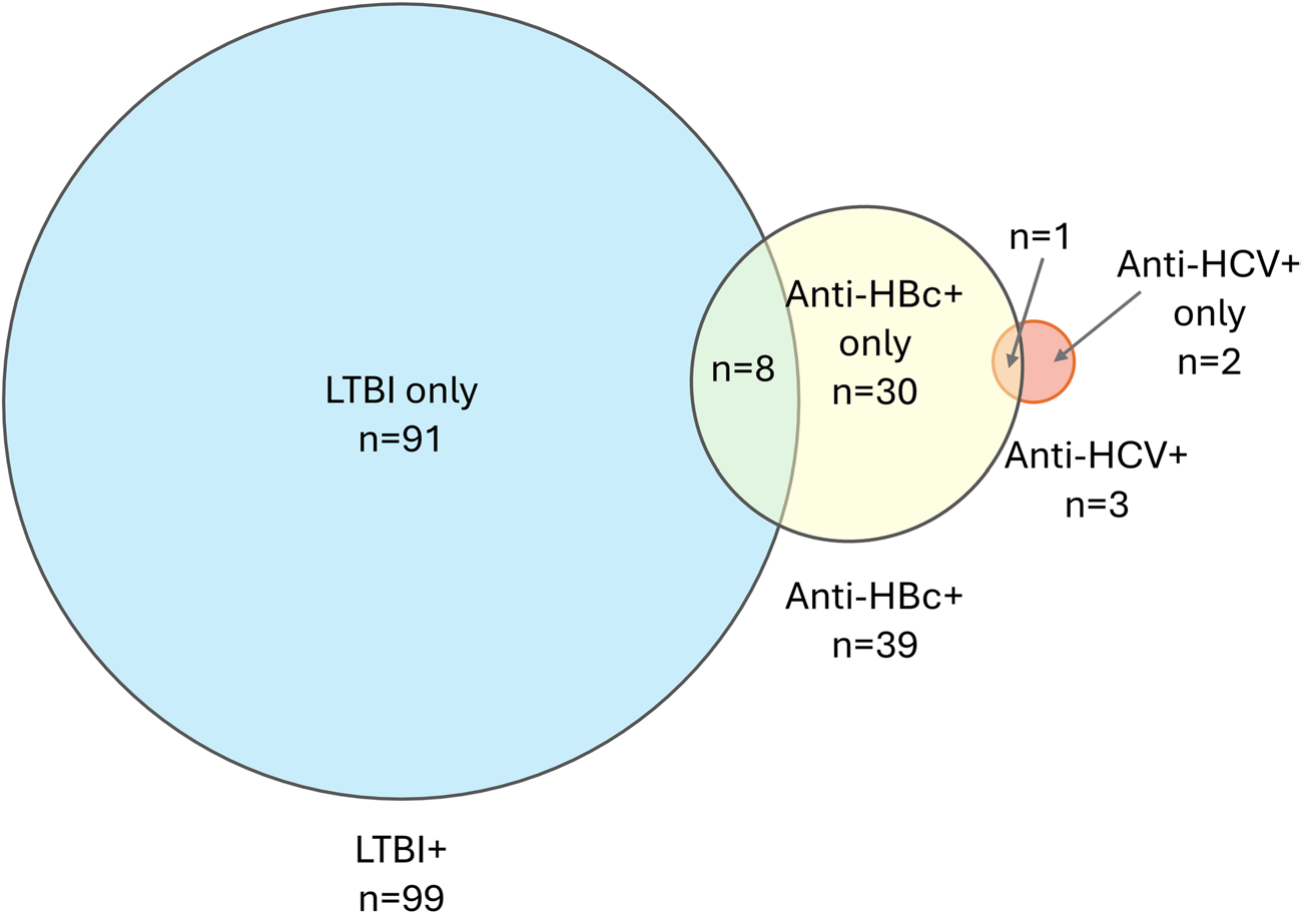
Venn diagram of positive results for LTBI, anti-HBc, and anti-HCV in the patient cohort (n = 697; anti-HBc data were missing for 30 patients and anti-HCV data were missing for 78 patients). HBc, hepatitis B virus core antigen; HCV, hepatitis C virus; LTBI, latent tuberculosis infection

Overall, 132 of 697 patients (18.9%) were positive for LTBI, anti-HBc, or anti-HCV (Table 2). LTBI was the most common positive result: 99 patients (14.2%) were positive for LTBI using any of the three assessments (combined patient report/history, QFT, or X-ray), while 39 (5.6%) were anti-HBc positive, and 3 (0.4%) were anti-HCV positive. Of the 39 patients who were anti-HBc positive, 8 (20.5%) were also positive for LTBI. One of the 3 patients positive for anti-HCV was also positive for anti-HBc; none was positive for LTBI (Fig. 1). There were no significant associations between positive tests for LTBI and anti-HBc, LTBI and anti-HCV, or anti-HBc and anti-HCV. None of the screened patients were identified with active TB. During the follow-up period, there were no confirmed cases of reactivation of TB based on patient records.

**Table 2:** LTBI, HBV, and HCV screening results (N = 697)

Screening test	n (%)
Positive on any (LTBI, anti-HBc, or anti-HCV)	132 (18.9%)
LTBI	
Any positive result	99 (14.2%)
QFT positive	59 (8.5%)
Positive on patient report and/or medical history^a^	42 (6.0%)
X-ray positive	17 (2.4%)
Anti-HBc	
Positive	39 (5.6%)
Negative	628 (90.1%)
Missing/unknown	30 (4.3%)
Anti-HCV	
Positive	3 (0.4%)
Negative	616 (88.4%)
Missing/unknown	78 (11.2%)

^a^17 (2.4%) had a positive patient self-report and 28 (4.0%; missing in 5 cases) had a positive medical history; 3 had both

HBc, hepatitis B virus core antigen; HBV, hepatitis B virus; HCV, hepatitis C virus; LTBI, latent tuberculosis infection; QFT, QuantiFERON

**Fig. 2:**
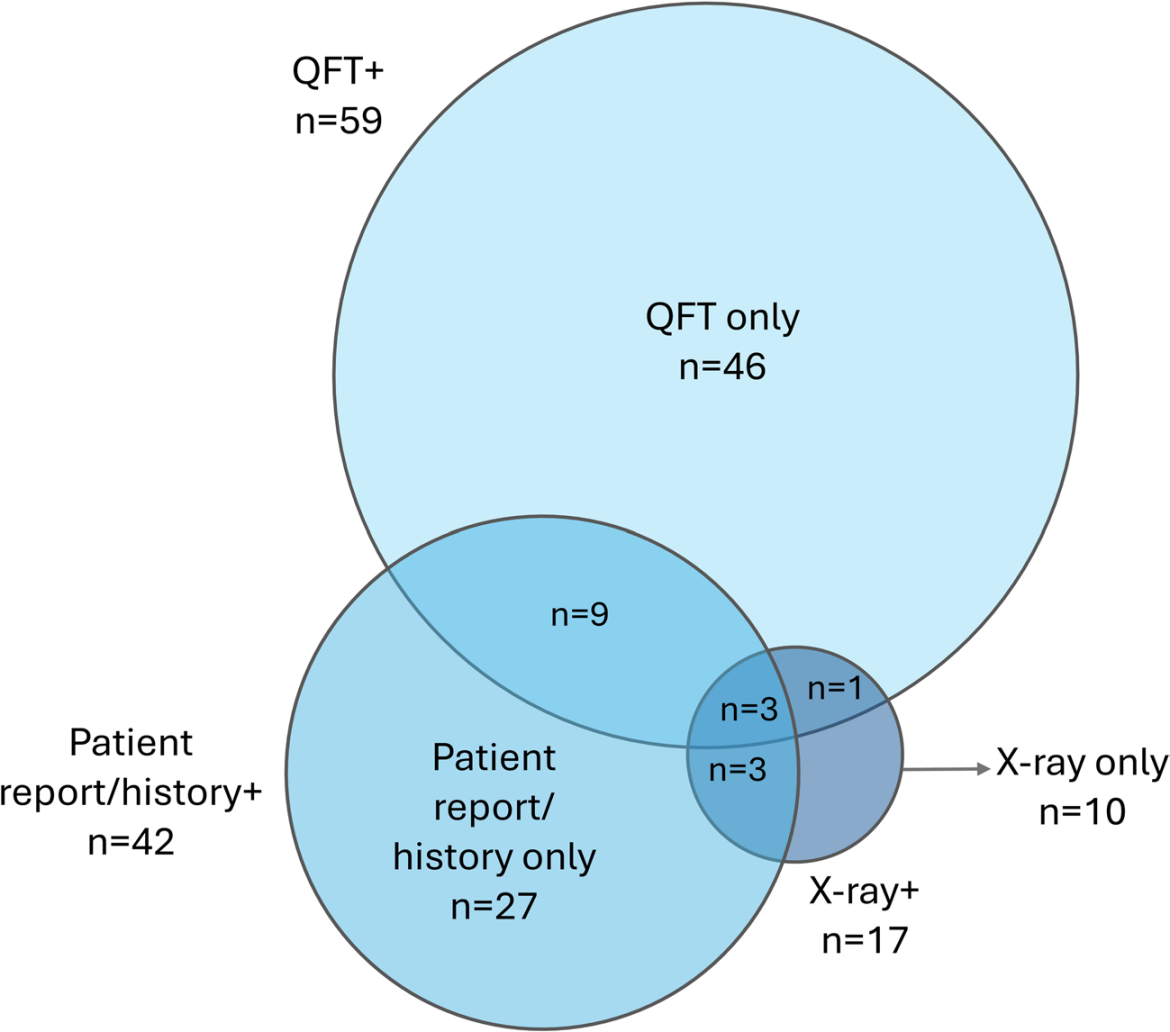
Venn diagram of positive results for LTBI testing. LTBI, latent tuberculosis infection; QFT, QuantiFERON. All patients (n = 697) were tested with all three assessments

QFT detected the most LTBI cases (59/99; 59.5%) and was the only positive test in 46 (46.5%) LTBI cases. Patient report/history identified 42/99 (42.4%) LTBI cases and was the only positive test in 27 (27.3%). Chest X-rays identified 17/99 (17.2%) LTBI cases and was the only positive test in 10 (10.1%). The overlap among the assessments was moderate (Fig. 2). Three patients (3.0%) were positive on all three tests, but most (83/99; 83.8%) were positive on only one of them. However, associations among all three tests were statistically significant: positive QFT results were significantly associated with patient report/history (12 patients; Fisher’s exact test two-sided p < 0.001) and abnormal X-rays (4 patients; p = 0.048), and patient report/history was also associated with abnormal X-rays (6 patients; p < 0.001).

The baseline characteristics of patients with a positive LTBI screening result were generally comparable to the overall patient cohort (Table 1), although patients with a positive LTBI test were slightly older (mean [SD] of 64.0 [13.8] vs 60.0 [15.8]) and had higher rates of RA (60.6% vs 55.5%). Six patients with a positive LTBI screening result (6.1%) reported that they had been vaccinated for TB.

### Subsequent therapy in screened patients 

The majority of patients (602; 86.4%) initiated intensified therapy with a bDMARD in the time period immediately after screening, 41 (5.9%) initiated tsDMARD treatment, and 54 (7.7%) did not subsequently receive a bDMARD or tsDMARD (including cases with high dose glucocorticoids or screenings conducted purely for diagnostic purposes, e.g. to investigate fever of unknown origin, or patients who declined b/tsDMARD therapy following the screening) (Table 3). The most commonly initiated bDMARD class was tumor necrosis factor inhibitors (TNFi) (422 [60.5%]), and adalimumab was the most frequently used agent (206 [29.6%]). Janus kinase inhibitors (JAKi) accounted for almost all of the tsDMARD use (39/41 [95.1%]) and baricitinib was the most frequently used JAKi (18/39 [46.2%]).

**Table 3:** Subsequent therapies for rheumatology patients undergoing screening prior to immunosuppressive therapy in the time period immediately following screening

Medication	n (%)	
	All patients (N = 697)	Patients with a positive LTBI screening result (N = 99)
No subsequent medication	54 (7.7%)	16 (16.2%)
bDMARD	602 (86.4%)	73 (73.7%)
TNFi	422 (60.5%)	45 (45.5%)
Adalimumab	206 (29.6%)	17 (17.2%)
Etanercept	131 (18.8%)	18 (18.2%)
Certolizumab	47 (6.7%)	5 (5.1%)
Golimumab	28 (4.0%)	4 (4.0%)
Infliximab	10 (1.4%)	1 (1.0%)
IL-6Ri	92 (13.2%)	14 (14.1%)
Tocilizumab	89 (12.8%)	14 (14.1%)
Sarilumab	3 (0.4%)	0
IL-17Ri	57 (8.2%)	9 (9.1%)
Secukinumab	57 (8.2%)	9 (9.1%)
Anti-B cell therapies	16 (2.4%)	4 (4.0%)
Rituximab	13 (1.9%)	4 (4.0%)
Belimumab	3 (0.4%)	0
CTLA4-Ig	12 (1.7%)	1 (1.0%)
Abatacept	12 (1.7%)	1 (1.0%)
Other^a^	3 (0.4%)	0
tsDMARD	41 (5.9%)	10 (10.1%)
JAKi	39 (5.6%)	8 (8.1%)
Baricitinib	18 (2.6%)	2 (2.0%)
Upadacitinib	11 (1.6%)	4 (4.0%)
Filgotinib	5 (0.7%)	1 (1.0%)
Tofacitinib	5 (0.7%)	1 (1.0%)
PDE4i	2 (0.3%)	2 (2.0%)
Apremilast	2 (0.3%)	2 (2.0%)

^a^Other bDMARD therapies were anakinra (n = 1), canakinumab (n = 1), and guselkumab (n = 1)

bDMARD, biologic disease-modifying antirheumatic drug; CTLA4-Ig, cytotoxic T lymphocyte-associated antigen-4-immunoglobulin; IL-6Ri, interleukin 6 receptor inhibitor; IL-17Ri, interleukin 17 receptor inhibitor; JAKi, Janus kinase inhibitor; LTBI, latent tuberculosis infection; PDE4i, phosphodiesterase 4 inhibitor; TNFi, tumor necrosis factor inhibitor; tsDMARD, targeted synthetic biologic disease-modifying antirheumatic drug

Patients with a positive LTBI screening result had higher rates of no subsequent medication intensification (16.2% vs 7.7%) and tsDMARD use (8.1% vs 5.6%) immediately following screening and lower rates of bDMARD use (73.7% vs 86.4%), particularly with TNFi (45.5% vs 60.5%). The rates of use of other bDMARDs, including interleukin (IL)−6 receptor inhibitors and IL-17 receptor inhibitors, were similar to rates observed in the full patient cohort (Table 3). There was a significant difference between the usage of b/tsDMARDs in patients without a positive LTBI screening result compared with those who did have a positive LTBI screening result (p = 0.031).

## Discussion

Our study evaluated the prevalence of positive screening tests indicating possible prior exposure to pathogens with the potential for reactivation during immunosuppressive therapy and found that a substantial proportion (18.9%) of rheumatology patients preparing to initiate intensified immunosuppressive therapy were positive for LTBI, anti-HBc, or anti-HCV, most frequently LTBI (14.2%). These results are particularly notable given that Germany is considered a low-prevalence country for these pathogens [19, 20] and indicate that rheumatologists should not become complacent about rigorous screening of their patients, even if they live in countries where the diseases are uncommon.

Screening guidelines for key pathogens in patients initiating immunosuppressive therapy are in place in Germany [7] and internationally [8–11, 21] but studies suggest these recommendations are not rigorously followed in some places. Although most rheumatology patients in Germany appear to be screened [22], studies in other locations have reported suboptimal screening rates ranging from 29% of patients with appropriate HBV testing in a multi-centre US study [23] to 65.5% for LTBI screening in the year prior to initiation of b/tsDMARD therapy in the American College of Rheumatology’s Rheumatology Informatics System for Effectiveness (RISE) registry [24]. The data from our study support the continued importance of screening to detect infections or prior exposures that may be reactivated during immunosuppressive therapy. Rescreening, especially during TNFi therapy, may also be appropriate in areas with a high TB prevalence [25].

The most common positive test in our screening was for LTBI, which was found in 14.2% of patients. This figure is similar to the estimated LTBI rates of 13.6% in Europe and 11.4% in the US, but higher than the estimated 7.9% rate of LTBI in Germany [26], possibly due to the increased risk of TB in rheumatologic patients compared with the general population [27]. Germany is considered a low-prevalence country for TB and BCG vaccination is not recommended [28]. However, as for many other developed countries with low rates of TB, recent influxes of immigrants have elevated the TB burden [15, 29–31], resulting in a heightened need for careful screening in patients preparing to initiate immunosuppressive therapy.

Over half the positive LTBI results (59/99; 59.6%) in our study were detected by QFT. However, all three assessments made important contributions to detecting LTBI, as the overlap among them was only moderate. For instance, chest X-ray, which produced the fewest positive results, was the only indication of LTBI in 10/17 cases. Other studies also support the importance of conducting chest X-rays in addition to IGRA tests when screening for LTBI [32, 33]. Our data provide strong support for the use of multiple types of assessments when evaluating potential LTBI in rheumatology patients rather than relying solely on a single assessment, such as QFT.

The second most common positive finding was a positive test for anti-HBc. Although HBV is a vaccine-preventable disease and is now included in the childhood vaccination schedule [28], vaccination rates in adults are often low; recent studies of German rheumatology patients have reported HBV vaccination rates of 7.8% to 12.5% [17, 34, 35]. The 5.6% anti-HBc-positive rate reported here is consistent with other studies in German rheumatology clinics [17, 34, 36] and similar to rates reported among patients on biologics in the US [37]. Although hepatitis B reactivation during b/tsDMARD therapy is relatively uncommon (2.0% in a recent meta-analysis), the risk during therapy is significant (odds ratio of 4.56) and higher with certain agents [38]. Anti-HCV results were rare in this cohort. Although the number of patients with a positive anti-HCV screening result was low, we consider these evaluations to be essential, as the identification of potential chronic infections with HBV or HCV allows the opportunity to treat the patient prior to initiating immunosuppressive therapy, ensure adequate prophylaxis, or monitor them closely during treatment.

There was minimal overlap between positive findings of LTBI, anti-HBc, and anti-HCV; very few patients had positive findings for more than one latent/chronic infection, and associations between positive results for LTBI and hepatitis viruses were not significant. However, our conclusions concerning overlap between HCV and HBV or LTBI are limited by the small number of anti-HCV-positive patients (n = 3).

Following patient screening, 92.3% of rheumatology patients initiated intensified immunotherapy, but this number fell to 83.8% in patients with a positive LTBI screening result. The use of bDMARD therapy was less common in the LTBI group compared with the overall patient cohort (73.7% vs 86.4%), primarily driven by lower rates of TNFi therapy (60.5% vs 45.5%). In contrast, tsDMARD use was higher in LTBI-positive patients compared with the overall cohort (10.1% vs 5.9%). Although these differences were statistically significant, the choice of therapy during the study period (2011 to 2024) was undoubtedly influenced by factors beyond the screening results. In particular, b/tsDMARD treatment patterns and availability of some drugs, particularly IL-6 receptor inhibitors, were impacted by the COVID-19 pandemic [39–41], and emerging evidence suggesting worse COVID outcomes in rheumatology patients treated with some agents, such as rituximab [42, 43], may also have modified treatment patterns. Of even greater importance, not all drugs were available throughout the entire period, resulting in time-dependent distribution of usage frequencies for different therapies. Drugs with new approvals may have had access restricted to after the use of other substances, been recommended as 2nd- or 3rd-line therapies in guideline-based treatment algorithms, or been used less frequently due to provider familiarity with more established agents. This may explain why tsDMARDs were infrequently employed as the first therapy following LTBI screening. Because of these various external factors, any observed associations between changes in treatments and LTBI positive screening results should be considered with caution.

It is not clear why patients with positive LTBI results had higher rates of tsDMARD use compared with the full patient cohort. Although some studies have reported lower rates of TB in RA patients treated with JAKi compared with bDMARDs [44, 45], an evaluation of patients with LTBI did not identify a difference in TB reactivation rates between JAKi and bDMARDs [44]. A higher TB reactivation rate was reported for JAKi compared with TNFi in one systematic review (1/11 [9.1%] vs 14/368 [3.8%]), but numbers in the JAKi group were very low and geographic variations in TB prevalence may have influenced these results [46]. At this point, there is no definitive evidence for a difference in LTBI reactivation rates in patients treated with tsDMARDs vs bDMARDS. The higher rates of tsDMARD use observed in LTBI patients in our study may therefore have related mainly to individual patient characteristics independent of the LTBI finding.

In addition to small numbers of anti-HCV-positive patients, other limitations of our study include a lack of information on exact assay values; our database listed results as positive and negative only, so it is unclear how many indeterminate results may have been included. Some patients did not have data for anti-HBc or anti-HCV, which may have affected the calculated positive test rates. We did not have access to additional HBV serological tests or HBV/HCV tests based on pathogen nucleic acid/viral loads; these were conducted on an individual basis as determined by the treating physician. Structured data for patients who were preparing to initiate treatment with high-dose glucocorticoids, including indication and planned dosage, were not available so we were unable to explore data in this subgroup of patients. Our database does not contain information on ethnicity or immigrant status; the LTBI burden is known to be higher in immigrant populations in Germany [29]. Data on HBV vaccination status was not collected in this study, but vaccination rates are likely similar to our previous report on HBV serology in this clinical setting [35].

## Conclusion

Our data indicate that almost one-fifth of rheumatology patients preparing to initiate intensified immunosuppressive therapy have positive results for LTBI, anti-HBc, or anti-HCV. Multiple types of assessments are required to detect all potential cases of LTBI. These findings support the need for careful screening in rheumatology clinics, including those in “low-prevalence” countries.

## Data Availability

The data underlying this article will be shared on reasonable request to the corresponding author.
